# Study protocol of personal characteristics and socio-cultural factors associated with mental health and quality of life of residents living in violent territories

**DOI:** 10.1186/s12888-020-02487-2

**Published:** 2020-03-03

**Authors:** Marcelo Santos Cruz, Eliana Sousa Silva, Miriam Krenzinger, Leandro Valiati, Dalcio Marinho Gonçalves, Maurício Teixeira Leite de Vasconcellos, Livia Melo Villar, Stefan Priebe, Paul Heritage

**Affiliations:** 1 Institute of Psychiatry, Federal University of Rio de Janeiro, Av. Venceslau Brás 71 fundos, Rio de Janeiro, 22290-140 Brazil; 2 Redes da Maré, R. Sargento Silva Nunes 1012. Nova Holanda, Maré, Rio de Janeiro, 21044-242 Brazil; 3 School of Social Work, Federal University of Rio de Janeiro, Av. Pasteur, 250, Urca, Rio de Janeiro, 22290-240 Brazil; 4 Federal University of Rio Grande do Sul. Faculdade de Ciências Econômicas, Anexo Av João Pessoa 52, Porto Alegre, Rio Grande do Sul 90040-000 Brazil; 5 Science, Sociedade para o Desenvolvimento da Pesquisa Científica. R. André Cavalcanti 81/301, Santa Teresa, Rio de Janeiro, 20231-050 Brazil; 6 Viral Hepatitis Laboratory of Oswaldo Cruz Foundation, Av Brasil, n° 4365, Manguinhos, Rio de Janeiro, 21040-900 Brazil; 7 Unit for Social and Community Psychiatry, Newham Centre for Mental Health, Queen Mary University of London, London, E13 8SP UK; 8 Department of Drama/People’s Palace Projects, c/o School of English and Drama, Queen Mary University of London, Mile End Road, London, E1 4NS UK

**Keywords:** Urban violence, Exposure, Public health, Mental disorders, Cross-disciplinary studies, Quality of life, Cultural practices, Coping, Cross sectional studies

## Abstract

**Background:**

Throughout the world, millions of people living in deprived urban environments with frequent experiences of violence are mentally distressed. There is little evidence about which characteristics of people living in such environments are associated with lower or higher levels of mental distress and how they may cope with experiences of violence.

**Methods/Design:**

This study is part of the research project ‘Building the Barricades’ (ES/S000720/1 ESRC-AHRC GCRF Mental Health 2017), which uses a mixed-method approach. Quantitative and qualitative studies will be conducted in 16 *favelas* in the area of Maré in Rio de Janeiro, Brazil. The quantitative study consists of a survey of 1200 randomly selected adults living in Maré and of 200 individuals who frequent the open-use drug sites. The survey will assess sociodemographic characteristics, experiences of different forms of violence, physical and mental health status (including drug use) and active participation in cultural consumption and production. In the qualitative study we will conduct 60 in-depth interviews and 8 focus groups of participants selected from respondents to the survey to assess in more detail their experiences of violence and coping strategies. In order to analyze the quantitative data we will use descriptive statistics and explore associations in uni- and multi-variable analyses. Qualitative data will be subjected to thematic analysis.

**Discussion:**

This is an exploratory study to identify characteristics and coping strategies that appear to help people to overcome experiences of violence in deprived areas without developing mental distress. The findings could inform policies to reduce mental distress and improve the quality of life of people living in urban areas affected by violence.

## Background

People living in urban neighbourhoods with low human development indices (LHDI) subject to frequent violence are vulnerable to the development of different types of mental disorders [1]. Global institutions like the Red Cross [2] and the United Nations Office on Drugs and Crime [3] have emphasized the need for strategies to deal with the humanitarian consequences of urban violence as it has been reported in various countries across all continents. Although there is evidence that some approaches, including mental health and psychosocial support, have improved emotional conditions and well-being [1], it is still unclear which are the best and more suitable interventions. Mental health and psychosocial support according to Tol et al. [1], includes ‘counselling, providing and facilitating community-based social supports, structured social activities (including child friendly spaces), provision of information, psychoeducation, and raising awareness’. The proposed study will provide new knowledge about the individual characteristics of people living in a particular urban environment with high levels of economic deprivation together with a socio-cultural analysis of the key features of that territory. The stated aim of the study is to identify strategies that enable residents to build and maintain their mental health resilience within a context of high levels of fatal conflict.

Wars, revolutions and insurrections are time-limited. Conflicts among armed criminal organisations and between armed criminal organisations and law enforcement agents are often more persistent, no matter what illicit product or activity is involved. In many of these scenarios, violence related to drug traffic and armed conflicts occur on a daily basis in densely inhabited environments [4]. Although there is no consensus concerning measures to investigate community violence, this concept is used in different studies including direct (i.e., victimization) and indirect experiences of public violence, i.e. witnessing different kinds of public violence, including shootings [5–7]. Community violence is also described as a ‘nondomestic, nonsexual and non-singular-event’ [7]. The expression ‘urban violence’ is also used in many studies with a similar meaning [8]. There is scant scientific information about the different types of violence that occur in these circumstances and most studies on violence focus on domestic or sexual violence or are directed towards specific parts of the population, such as violence motivated by racism or homophobia [9].

Since the 1980s, there have been reports of a progressive increase in morbidity and mortality as a consequence of violence in Brazil, mainly among non-white men aged 20 to 29 years with low socio-economic status [10]. In Brazil, urban violence related to drug/firearm traffic and its repression has been increasing. In 2016, there were 62,517 homicides [11], which is the highest absolute number per country in the world. As in many other countries, people living in deprived and urban neighbourhoods in Brazil are particularly and frequently exposed to violence [12]. The Complexo da Maré in Rio de Janeiro, Brazil, is one of these poor and violent environments. Maré is made up of 16 *favelas* and has more than 144,000 residents living in 47,000 households. There are 44 elementary schools, 3 high schools, 16 Residents’ Associations, 14 Non-Governmental Organisations (NGOs), 4 cultural centres, 3182 commercial enterprises, 10 health facilities, more than 90 churches, 1 Citizen Centre and 1 Police Battalion. In Maré, three different heavily armed organized crime groups control drug traffic and other businesses.

Redes da Maré [Maré Networks] has been developing a range of interconnected community-based activities for more than 20 years, including providing assistance and advice to drug users and people living on the streets. Redes da Maré also develops monitoring and analysis of armed confrontations, publishing an annual report on the *Right to Public Security in Maré* [13]. This report records police operations, military occupations, clashes between armed groups, homicides, ‘deaths by interventions of State agents’, shootings, injuries, closure of public facilities such as schools and health facilities, closure of private establishments, damage to property, theft of belongings / extortion, and emotional / psychological harms such as trespassing, physical and verbal abuse, torture, loss of family and friends, false imprisonment and restriction of mobility (the right to come and go).

A qualitative study in Maré showed that residents report a range of experiences that includes the conflicts between the heavily armed criminal organisations and between those organisations and the police [14]. Although there is officially one battalion of the military police that is in charge of the security of the area, police are neither regularly present nor active inside the community. Therefore, the “drug dealer’s law” is imposed on everyone. Police are only present when they “invade” the community during special operations. Crossfire that occurs during incursions by the police are responsible for many of the casualties and much of the chronic emotional stress. The territory and its residents are subject to multiple aggressions, either physical or psychological, that involve coercion and homicides both by the armed criminal organisations and by the police [14].

Individual characteristics such as gender, age, socioeconomic status, satisfaction with weight, diet, regular practices of physical activities, smoking, chronic disease have been reported as being associated with their perception of violence within neighbourhoods and with a self-evaluation of health and wellbeing [15]. Psychological characteristics are also associated with resilience to experiences of urban violence [16]. On the other hand, alcohol abuse, gender and low economic status have been described as associated with psychological distress after direct or indirect exposure to violence [17].

In the scientific literature, we did not find any household survey about the individual characteristics of people living in neighbourhoods affected by violence and socio-economic deprivation that took into account a range of variables such as quality of life, mental health indicators, cultural practices, drug-use patterns, experiences of violence and high-risk behaviours associated with blood and/or sexually transmitted diseases.

The present study has been co-created by academics and community leaders, and the preparations included meetings with residents to discuss the aims, research methods and procedures. Identification of the individual characteristics of people living in this particular urban environment with its high levels of economic deprivation and frequent experiences of violence as well as the socio-cultural features of that territory will provide information on resilience, protective and risk factors in the development of mental distress and the possibility of identifying collective and individual intervention strategies. Although the territory of Maré has its peculiarities, we can assume that the findings will have relevance in other metropolitan communities with low HDI measures in Latin America and the Global South, especially in urban territories affected by high levels of violence.

### Study questions

The overall aim of the research is to explore factors that support people living in neighbourhoods affected by violence and socio-economic deprivation to avoid or reduce mental distress. The specific research questions are 1) What experiences of violence do residents at Maré report, and which personal characteristics are associated with the experience of violence? 2) How are experiences of violence perceived and reported by residents in Maré and what are their strategies for coping with them? 3) Is there an association between experience of violence and the resident’s mental health and quality of life? 4) Which personal characteristics and socio-cultural factors are associated with more or less favourable mental health and quality of life of residents living in territories affected by high levels of violence and socio-economic deprivation?

## Methods

### Study status

Study was in Recruitment phase at the moment of the article’s submission.

### Study design

This study is part of the research project *Building the barricades: interdisciplinary studies on Mental Health, Wellbeing and Substance Use Disorders in the context of armed violence in Brazil*(ES/S000720/1 ESRC-AHRC GCRF Mental Health 2017) which comprises broader objectives, including mapping community network services and introducing arts-based interventions. The exploratory study will use a mixed-method approach, combining quantitative and qualitative research techniques. The quantitative study is a cross-sectional observational study of 1400 interviews of individuals who live in the favelas in the area of Maré in Rio de Janeiro, Brazil. The qualitative part will include 60 in-depth interviews and 8 focus groups with participants selected during the quantitative interviews.

### Participants

Adults who live in Maré will be invited to participate by providing information about the aims, content and methods of the study. More than 144,000 residents live in low-quality housing stock (with an average of 3.1 individuals per house) but most of them have regular or temporary work. The residents of Maré have a life expectancy of 66.6 years and the community has one of the lowest Human Development Indices (0.722) among all boroughs of Rio de Janeiro. There are 4 open-use drug sites in the community and they are frequented by around 200 individuals at any time. Most of them are using crack and/or alcohol, taking up ‘residence’ in these sites temporarily (for days) or permanently.

The quantitative study will include a household survey of 1200 individuals who live in Maré plus 200 individuals who are a regular part of the open-use drug scene.

### Measures

1. Sociodemographic profile: age, gender, marital status, highest completed education level, ethnicity, employment, accommodation, living situation (alone or not).
2. Access to culture and to social and health services: self-reported (a) frequency of different forms of cultural participation (including watching movies, going to the theatre, listening to music, reading books, watching TV, going to a museum, dancing, acting, singing, playing music, writing, painting, photographing, etc); (b) physical and mental problems and (c) frequency of utilization of health services; (d) existence and frequency of impediment of utilization of social, health and cultural activities because of experiences of violence.
3. Mental health profile: Levels of mental distress, measured by the Brief Symptoms Inventory (BSI) [18].
4. Subjective quality of life, measured by the Manchester Short Assessment of Quality of Life [19].
5. Drug use pattern: (a) Utilization of legal and illicit drugs in the past 3 months, (b) urges, (c) problems, (d) attempts to control use and (e) injected utilization measured by Alcohol, Smoking and Substance Screening Test (ASSIST) [20].
6. Experiences of violence, measured as: self-reported episodes of (a) direct violence (b) indirect violence exposure, and (c) harmful consequences of violence exposure.
7. Behaviours that may be associated with blood and/or sexually transmitted diseases. Self-report of (a) unprotected sex, (b) sharing paraphernalia for drug injection or smoking, (c) tattoo or (d) piercing.

### Instruments

For the quantitative part we will use a questionnaire designed specifically for this study. It will include 7 sections, each one directed to the measures described above. The sections are based on and partly include other instruments:

1. Sociodemographic profile: Based on the Brazilian Institute of Geography and Statistics questionnaire adapted to specific local situation.
2. Access to culture and to social and health services: Developed specifically for the study, based on the set of services available in the area of Maré and on information provided by residents of Maré about existing social and health services as well cultural activities provided by formal and informal institutions/organisations.
3. Mental health profile. Brief Symptoms Inventory (BSI) [18]. The Brief Symptoms Inventory has 53 items and evaluates 9 domains (somatization, obsessive-compulsion, interpersonal sensitivity, depression, anxiety, hostility, phobic anxiety, paranoid ideation and Psychoticism) and three global indices: global severity index, positive symptom stress index, and positive symptoms total. The global indices measure current or past level of symptomatology, intensity of symptoms, and number of reported symptoms, respectively. It is translated and validated into Portuguese and the application time is 8–12 min [21].
4. Manchester Short Assessment of Quality of Life (MANSA) [19]. MANSA is an instrument for assessment of quality of life. It has 24 questions and provides scores for quality of life.
5. Alcohol, Smoking and Substance Screening Test (ASSIST). ASSIST is a screening instrument for detection of substance use. It was developed by World Health Organisation and translated to Portuguese and validated [20]. It includes 8 questions about existence of use of 10 different categories of legal and illicit drugs in the past 3 months, urges, problems, attempts to control use and injected utilization.
6. Experiences of violence: Developed specifically for the study, based on the data of the Bulletin *The Right to Public Security in Maré* [13], and also on the reports of Maré residents and professionals. This session also includes questions from the Addiction Severity Index [22]. The experiences investigated include direct (e.g. being beaten, menaced) and indirect exposure (e.g. seeing someone been beaten or killed), and harmful consequences of violence exposure (e.g. being unable to attend school or health care consultation, feeling fear or mental distress).
7. Behaviours that may be associated with blood and/or sexually transmitted diseases: Includes unprotected sex, sharing paraphernalia for drug injection or smoking, tattoo or piercing.

### Interviewers training and data collection quality

Eight interviewers were selected for quantitative and six for qualitative phases. Selection was based in the candidate’s experiences on research data collection. For quantitative phase, training of the interviewers on the use of the questionnaire and tablets was conducted by the study coordinators and a pilot study with 20 interviews was done. For qualitative phase, training with the topic-guide in-depth and focus group interviews was performed. Study coordinators Data Monitoring Committee will perform weekly meetings for supervision of interviewers and for auditing all study activities conduct and data quality monitoring.

Household survey: 1200 individuals. Respondents must have lived in a house in the territory of Maré during the past 3 months (living in a house in Maré is defined as having slept or spending most of the nights or days in a house during the 3 months prior to the interview). The household survey will not include any respondents who frequent the open-use drug sites.

Participants who frequent the open-use drug sites: 200 individuals who have frequented and taken drugs at the open drug-use sites in Maré during the 3 months prior to the interview.

### Inclusion and exclusion criteria

#### Inclusion criteria

People will be 18 years of age or older and provide written informed consent to participate.

#### Exclusion criteria

People will be excluded if they lack capacity to consent, refuse or withdraw consent, or have cognitive impairments severe enough to prevent them providing information on the study instruments.

### Geographical stratification

The sample was defined to take into account three geographic strata composed of contiguous communities (favelas) in the territory of Maré that share residential characteristics and similar social dynamics, as well as public roads and public facilities. Strata 1, 2 and 3 cover four, nine and three *favelas*, respectively.

### Sample size determination

Considering that there is no previous information about the prevalence of the main variables studied in the survey population, we choose the minimum proportion Pmin = 3% for which the relative error of the estimation should be at most dR = 60%, which corresponds to a relative margin of error of 1.8%, with a confidence level of 100 × (1-α) = 95%.

According to Cochran [23] and assuming simple random sampling (SRS) without replacement, the sample size needed to estimate proportions equal to or greater than Pmin with relative error not greater than dR at the 1-α confidence level is given by: ${\mathrm{n}}_{\mathrm{SRS}}=\frac{{\mathrm{z}}_{\upalpha /2}^2}{{\mathrm{d}}_{\mathrm{R}}^2}\times \frac{1-{\mathrm{P}}_{\mathrm{min}}}{{\mathrm{P}}_{\mathrm{min}}}$.

From the above expression and with the aforementioned parameters, the minimum sample per stratum should be 346 individuals. Since only one person per household will be selected, the sample size of households will be the same. As the geographic strata indicated above are also controlled-precision estimation domains, it was decided to use the sample size of 400 people per stratum, resulting in a survey of 1200 people (or households) in the total sample.

### Selection methods

The sample will be selected based on the Address Register generated during the collection of the Maré Census 2013 [24], conducted by Redes da Maré in partnership with the Observatório de Favelas. The size of the sample is based on the same survey.

An inverse sampling procedure will be applied to the Address Register, taking into account the geographical strata. Thus, the use of the inverse sampling will result in the selection of addresses which must be visited in order to interview each resident. The household sequential visiting procedure will be ended when the predetermined number of completed interviews has been reached [25–27].

The selection of the resident to be interviewed in each household will be done according to the eligible residents using equal probabilities, i.e. a simple random sampling. If the selected person is absent, the household will receive up to two extra visits, at different days and times, to obtain the interview. If it is not possible to interview the selected resident, the sequential procedure (or the sequential address to be visited) will continue with the next address in the random order of visits.

The inverse sample method as replacement strategy does not bring bias to the sample and is better than increasing the sample size to account for non-response, since non-response rates are different according to the characteristic of each area and other variables.

### Open-use drug sites sample

In Maré there are 4 different drug-use scenes. According to the regular registers of Redes da Maré outreach work, the total number of individuals who frequent these sites varies but is approximately 200 people. So, the subsample size of individuals who frequent the drug scene will include the whole population of those areas.

### Procedures

#### Household survey

The field researchers will be identified by the wearing of a t-shirt and an official badge/document from Redes da Maré. When arriving at the selected addresses indicated on the collection sheet, the field researcher will (a) explain the purpose of the research, (b) guarantee the anonymity of the information, indicating that the questionnaire will not be nominative and will only be identified by a number (c) check the inclusion / exclusion criteria, (d) inform the average time of application of the questionnaire (60 min), and finally (e) invite the participant to be interviewed. After agreement has been demonstrated by signed consent, the interview will be carried out inside the home, in a place that guarantees privacy. Data will be collected directly on portable electronic devices (“tablets”). The households will be visited sequentially by each field researcher until the quotas are completed, as predicted by the reverse sampling. Home field research will take place over 6 months through the application of 10 questionnaires per day (20 working days per month).

#### The open-use drug site

Users of the site will be invited to participate by outreach workers. Four participants per day will be invited to participate by the recruiters and will be accompanied by them to the interview. The place of application of the questionnaire is located 10 min’ walking distance from the site of open-drug use frequented by the respondents. The interviews will occur in a Drop-in Centre that receives daily guests from the open-use site. It has a service room on the second floor suitable for the application of the questionnaire, ensuring privacy and a welcoming environment.

Upon arrival in the Drop-in Centre, all those invited to participate who meet the inclusion / exclusion criteria and provide informed consent will be interviewed in a reserved room. Interviews will be conducted by trained interviewers with the questionnaire. Answers to closed questions and the rapid test result will be recorded on hand-held computer tablets. After the interview, the participant will receive a meal voucher as compensation for the time spent in the interview. The expected duration of the quantitative interview is 60 min. Open Drug-use site frequenters interviews will take place over 3 months through the application of 8 questionnaires per day (8 working days per month).

### Qualitative assessment

#### Qualitative phase interviews and focus groups

The qualitative phase will include 60 in-depth interviews and 8 focus groups.

In-depth interviews: 30 interviews will be conducted with individuals residing in households and 30 with those who frequent the drug use scenes.

### Selection and recruitment of potential participants in qualitative phase interviews

During the quantitative interviews for both the in-depth interviews to be held at respondents’ homes and those with the frequenters of the open-use drug sites, the interviewers will select and invite potential participants for the qualitative phase. The interviewers will select participants who report distinct aspects with the aim to cover an ample spectrum of personal characteristics and experiences. Participants of both sexes, distinct age groups, of the three geographic strata, different levels of education and of different religions will be invited. We will also invite potential participants who report frequent and non-frequent engagement with cultural practices, who have varying experiences of violence, are using and not using crack and display different levels of mental distress.

The interviewees for the qualitative study will be selected by purposive sampling with the following criteria:

- Both sexes, 18 years and older
- Self-reported experience of violence in the past 12 months
- Self-reported intense or low levels of participation in cultural practices
- Self-reported higher or lower levels of mental distress
- Self-reported higher or lower levels of drug use

### Procedures for in-depth interviews

The invitations and appointments will be made by the team of field researchers. The interviews will be held in secure and private spaces at the main headquarters of Redes da Maré. Upon arrival, all those invited to participate who manifest agreement to participate signing the Informed Consent Form will be interviewed in a reserved room. All in-depth interviews will be conducted by interviewers experienced in qualitative research techniques and will be topic-guided. The topic guide has been developed in discussions between the research team, residents and community leaders. It makes use of information provided by NGO workers in Mare including the data reported in the Bulletin *Right to Public Security in Maré* [13]. The topic guide will be piloted and amended if necessary. The topics will focus on the residents’ experiences of violence, how they deal with these situations and what strategies are used to deal with the context. As support, the material will be recorded for later transcription of the most relevant data. After the interview, the participant will receive a meal voucher as compensation for the time spent in the interview. The expected duration of the qualitative interview is 120 min. In-depths and focus groups interviews will take place over 3 months.

### Focus groups

For the focus groups, approximately 60 participants will be heard, divided into eight groups (6 to 8 participants per group).

### Recruitment

Focus group participants will be nominated and invited by field researchers using the same criteria used to select the participants of the in-depth interviews.

### Focus group procedures

The researchers / facilitators of the eight groups will meet at the headquarters of Redes da Maré, for 2 h. The aim is to collect information about key research topics from the dialogues and debate that take place in the focus groups. The topics will be focused on the experiences of violence, and what strategies participants use to deal with specific experiences in the context of Maré. The guiding questions of the study will nourish the debate among the participants, without trying to achieve consensuses. The focus groups will be conducted by two trained facilitators. One will conduct the discussion and a second one will record and annotate the lines, naming them and associating them with the motives that incited them, emphasizing the ideas contained therein. The reporter will also be asked to record the nonverbal language of the participants, such as voice tones, facial expressions and gesticulation. As support, the material will be recorded for later transcription of the most relevant data. After the interview, participants will receive a meal voucher as compensation for the time spent in the interview.

### Statistical analyses

Descriptive analyses will be undertaken to characterize the profile of the population of the study with distribution by different variables. Bivariate analyses will be conducted aiming at observing differences in prevalence between the groups according to the variables of interest including experiences of violence, mental distress, quality of life, cultural and social practices. To this end, the chi-square test and Fisher’s exact test (when the expected value was less than five) will be used. Where possible multivariable analyses will be conducted. Differences and associations will be considered significant at *p*-value ≤0.05. The data will be processed and exploited in Microsoft Excel and IBM SPSS programs.

### Qualitative analyses

The material collected through in-depth interviews and focus groups will be analysed through in-depth qualitative method analysis procedures (eg. thematic / narrative). All recorded interviews will be transcribed, and each participant will be identified by letters and numbers. The data transcribed will be analyzed with the software NVIVO in three steps: 1) extensive reading of all the transcribed data by two different researchers, 2) creation of codes to name each segment of information, 3) examining each code created in step 1, selecting the most frequent and significant then integrating, synthetizing and organizing them in categories and subcategories. Discrepancies of judgment of the two researchers will be discussed and decided with a third researcher. The analyses will consider literature theories and hypotheses about exposure to urban violence and social and cultural experiences.

The triangulation of the data found with both methods will aim to achieve the specific and main objectives of this research.

### Ethics approval

The protocol was submitted and approved by Brazilian National Commission for Research Ethics under the number CAAE: 01944918.2.0000.5263.

### Dissemination

The research findings will be disseminated through various means that aim to contribute to the development of public policies and community interventions/strategies that empower people living in LHDI urban neighbourhoods affected by high levels of armed violence. The intended means of dissemination include the publication of articles in peer reviewed scientific journals, scientific reports and presentation at congresses and other scientific meetings. The results will be discussed in meetings with community leaders and residents and with senior leaders of local and national cultural, social and health care networks. The publication of a report describing the study process and results is also previewed in the study project. We hope that the dissemination of the study will contribute to Brazil’s and other countries’ efforts to achieve objective 3 of the Sustainable Development Objectives as defined by the World Health Organisation: Ensure healthy lives and promote wellbeing for all at all ages.

## Discussion

The present study will provide robust information about the distribution of different types of violence affecting people living in LHDI and urban neighbourhoods affected by high levels of armed conflict. It will provide a sociodemographic profile, which includes cultural, social and health practices, mental distress, drug-use patterns and behaviours that may be associated with blood and/or sexually transmitted diseases. The proposed study presents some challenges. It will provide data about the mental health of what we believe to be the largest sample of people living within an urban territory not in a war zone but affected by persistently high levels of armed conflict. Objective research methods, procedures and themes of investigation have been co-created by the research team with local residents and community leaders. The present study will complement information on violence provided by studies that focus on domestic, sexual, child-related, war-related or other time-limited issues. The proposed study will also add relevant information that may assist the construction of strategies to cope with the violent environment. As emphasized by Tol et al. [1] the gap between research and practices directed to cope with this kind of violence needs to be reduced to make possible the identification and development of successful interventions to provide mental health and psychosocial support. Many studies about interventions are focused on the prevention of violent behaviours. It is obvious that the ideal is to avoid or reduce levels of violence, but this often depends on complex political or economic changes. Until steps to decrease or avoid urban violence are successful, the development of strategies to cope with persistent situations is necessary. It is necessary to find strategies that focus on actions that are within the reach of those living in these communities and that consider the individual characteristics of those affected by violence. For that reason, the proposed data collection about personal characteristics may illuminate whether some of these attributes are related to a better or worse mental status, providing indications for future development of interventions to increase coping resources.

Although urban violence in Brazil may be similar to what happens in other low-to-middle-income countries as well as in some high-income countries, there are obvious local peculiarities. For that reason, generalizations to similar contexts in other countries and continents require careful consideration. The nature of the study - a cross-sectional observation - does not provide evidence for causal relationships. Nevertheless, if the present study shows that individuals with more cultural and social activities have lower levels of mental distress, drug-use patterns and/or less risk behaviours, the proposed study may lead to new research questions and have implications for policies to promote access to such activities. This is even more relevant, considering that experimental study exposing people to urban violence in a controlled way is out of the question.

The study addresses a general problem of increasing urbanization and may advance our understanding of violence and mental distress focusing not only on deficits, but also on the strengths of people in adverse contexts. The involvement of people from Maré will contribute to the validity of the study and empower the residents. The proposed study is also a step towards rigorous research going beyond mere assumptions and opinions and contributes to filling current gaps in knowledge through stringent research conducted in some of the most disadvantaged areas in the world.

## Supplementary information

**Additional file 1.** In-depth interview script and focus group guidelines. Translated copy of the Questionnaire developed to the Qualitative interviews.

## Data Availability

The datasets used and/or analyzed during the current study are available from the corresponding author on reasonable request.

## Abbreviations

AHRC: Arts and Humanities Research Council
ASSIST: Alcohol, Smoking and Substance Screening Test
BSI: Brief Symptoms Inventory
CAAE: Certificado de Apresentação para Apreciação Ética (Presentation Certificate for Ethical Appreciation)
ESRC: Economic and Social Research Council
GCRF: Global Challenges Research Fund
IBM SPSS: International Business Machines Corporation Statistical Package for the Social Sciences
LHDI: Low human development index
MANSA: Manchester Short Assessment of Quality of Life
NGO: Non-governmental Organisation
SRS: Simple random sampling
UNODC: United Nations Office on Drugs and Crime
