# Supplementary material for: Study protocol of personal characteristics and socio-cultural factors associated with mental health and quality of life of residents living in violent territories
Source: BMC Psychiatry. 2020 Mar 3;20:96. doi: 10.1186/s12888-020-02487-2 (PMC7055079; doi:10.1186/s12888-020-02487-2)
Supplement: Supplementary file 1 — Additional file 1. In-depth interview script and focus group guidelines. Translated copy of the Questionnaire developed to the Qualitative interviews. [file 12888_2020_2487_MOESM1_ESM.doc]

**In-depth interview script and focus group guidelines**

- What is your perception on the impact of armed conflicts on your mental health and well-being, as well as among your family members living in the favelas of Maré, Rio de Janeiro?
- Which public policies and local services and protection networks are being used by adult men and women living with mental disorders or social suffering in the communities of Maré?
- Does any socio-cultural activities existing within the Maré community contribute to your physical and mental health and well-being? In what way?
- What community resources, based on family support, civil society organizations, social and cultural organizations do you use and what do you think of them?
- Which strategies offered by the Maré intersectoral network of services and local agencies do you make use to contribute to your mental health?
- What strategies do you use to cope/deal with situations of violence and stress after experiencing a situation which involves violence?
- How would you describe your access to social, health and cultural services? Do you find it difficult to access these services? If so, which of them?
- Do you find it difficult to access services due to situations of violence? How do you get around these difficulties?
- Do access to these services contribute to the promotion of your physical and mental health? How?
- How do you deal with mental health disorders (yours or your family members)?
